# Oral uracil–tegafur compared with intravenous chemotherapy as adjuvant therapy for resected early‐stage non‐small cell lung cancer patients

**DOI:** 10.1002/cam4.6440

**Published:** 2023-08-09

**Authors:** Sheng‐Kai Liang, Chang‐Wei Wu, Ching‐I Chang, Li‐Ta Keng, Meng‐Rui Lee, Jann‐Yuan Wang, Jen‐Chung Ko, Wei‐Yu Liao, Kuan‐Yu Chen, Chao‐Chi Ho, Jin‐Yuan Shih, Chong‐Jen Yu

**Affiliations:** ^1^ Department of Medicine National Taiwan University Cancer Center Taipei Taiwan; ^2^ Department of Internal Medicine National Taiwan University Hospital, Hsinchu Branch Hsinchu Taiwan; ^3^ Department of Nursing, National Taiwan University Hospital and School of Nursing, College of Medicine National Taiwan University Taipei Taiwan; ^4^ Department of Internal Medicine, National Taiwan University Hospital and College of Medicine National Taiwan University Taipei Taiwan

**Keywords:** adjuvant therapy, chemotherapy, early‐stage NSCLC, uracil–tegafur

## Abstract

**Background:**

Studies comparing the effectiveness of either adjuvant oral uracil‐tegafur (UFT) or intravenous chemotherapy on early‐stage (stage I and II) non‐small cell lung cancer (NSCLC) patients treated with complete surgical treatment remain limited.

**Methods:**

From January 2011 to December 2017, patients with early‐stage NSCLC (defined as tumor size >3 cm without mediastinal lymph node involvement or any distant metastasis) receiving either adjuvant oral UFT or intravenous chemotherapy after surgical resection were identified from the Taiwan Cancer Registry. Overall survival (OS) and relapse‐free survival (RFS) were the primary and secondary outcomes, respectively. Propensity matching was used for controlling confounders.

**Results:**

A total of 840 patients receiving adjuvant therapy after surgery (including 595 oral UFT and 245 intravenous chemotherapy) were enrolled. Before matching, patients using oral UFT had significantly longer OS (HR: 0.69, 95% CI: 0.49–0.98, *p* = 0.0387) and RFS (HR: 0.79, 95% CI: 0.61–0.97, *p* = 0.0392) than those with intravenous chemotherapy. A matched cohort of 352 patients was created using 1:1 propensity score‐matching. In the Cox regression analysis, the UFT and the matched chemotherapy groups had similar OS (HR: 0.80, 95% CI: 0.48–1.32, *p* = 0.3753) and RFS (HR: 0.98, 95% CI: 0.72–1.34, *p* = 0.9149). Among subgroup analysis, oral UFT use was associated with longer RFS among the subgroups of non‐drinker (HR: 0.66, 95% CI: 0.34–0.99, *p* = 0.0478) and patients with stage IB disease (HR: 0.67, 95% CI: 0.42–0.97, *p* = 0.0341).

**Conclusions:**

This population‐based study in the real‐world setting of Taiwan demonstrates comparable effectiveness between oral UFT and intravenous chemotherapy in terms of clinical outcomes for early‐stage NSCLC patients after surgery.

## Graphical abstract

Adjuvant uracil‐tegafur (UFT) for early‐stage resected non‐small cell lung cancer (NSCLC) patients may be as effective as chemotherapy. Oral UFT could be an alternative option as adjuvant therapy beyond current guideline.

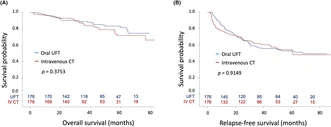

## 1 INTRODUCTION

Lung cancer has been the most common cause of all malignancy‐related deaths for years, despite the great advances in lung cancer treatment we have had over the past two decades.^1^ Utilization of low‐dose computed tomography as a screening tool for either the high‐risk population or never smokers with lung cancer family history could help reduce lung cancer mortality, and further curative surgery could be applied for those early identified patients with stage I and II non‐small cell lung cancer (NSCLC).^2^, ^3^ Although definitive surgery is standard treatment strategy for stage I to IIIA resectable NSCLC as a curative management, tumor recurrence will be the most important factor influencing patient's long‐term survival.^4^ Therefore, adjuvant chemotherapy or chemoradiotherapy is rationally applied in patients with early‐stage NSCLC after definitive surgery, and these adjuvant therapies are expected to reduce the recurrence rate and prolong survival for those patients with early‐stage resected NSCLC.^5^, ^6^

The 5‐year survival rate of all patients with stage I NSCLC rapidly declines from more than 80% for those with pathologic stage IA disease to less than 70% for those with stage IB disease,^4^, ^7^, ^8^ but there is no greater benefit for overall survival (OS) in patients with stage I NSCLC receiving adjuvant chemotherapy after curative surgery than in those receiving surgery alone.^9^ However, some studies found that adjuvant chemotherapy could provide an OS benefit in patients with stage I NSCLC with a tumor size 4 cm or larger in diameter by subgroup analysis,^9^ and administration of adjuvant chemotherapy could have a clinical benefit for those patients with early‐stage NSCLC and visceral pleural invasion (VPI), which was associated with high recurrence rate and low 5‐year OS rate.^10^, ^11^, ^12^ According to the National Comprehensive Cancer Network (NCCN) guideline for lung cancer management, adjuvant chemotherapy was recommended for patients with high‐risk T2 tumors without regional lymph node involvement (N0) NSCLC, including those who were without R0 resection, had poorly differentiated tumors, underwent wedge resection, had tumor larger than 4 cm, and had tumor with VPI.^13^ Therefore, patients with early‐stage NSCLC having the abovementioned risk factors for tumor recurrence are considered to benefit from adjuvant chemotherapy after curative surgery.

Despite the improvement in 5‐year OS and quality‐adjusted survival in patients with postoperative early‐stage NSCLC treated with chemotherapy as adjuvant therapy,^14^, ^15^ low treatment compliance due to conventional chemotherapy‐related toxicities, such as neutropenia and asthenia, is still the major concern in clinical practice.^16^ The combination of uracil‐tegafur (UFT) is an oral form chemotherapy while tegafur, a prodrug, is converted to 5‐fluorouracil (5‐FU) as a broad antitumor drug, and uracil inhibits dihydropyrimidine dehydrogenase to increase 5‐FU plasma concentration and then enhance anticancer activity.^17^, ^18^ Additionally, a study from the Japan Lung Cancer Research Group indicated that oral UFT as a single adjuvant therapy could reduce the mortality risk by 52% in patients with NSCLC with T2 disease after surgical treatment,^19^ and other meta‐analysis results also showed that adding UFT use significantly prolonged survival time in patients with post‐operative stage I NSCLC compared with surgery alone.^20^, ^21^ Meanwhile, around 2% of patients using oral UFT experienced grade 3 toxic events but no grade 4 adverse reactions,^19^ and tolerable drug‐related toxicities for patients could help improve therapeutic compliance. Although UFT is not effective as monotherapy for NSCLC treatment because of the low response rate (6%–8%),^19^, ^22^, ^23^ the application of oral UFT as adjuvant chemotherapy in patients with early‐stage NSCLC is used clinically in Japan and Taiwan.^19^

Currently, oral UFT is not included as the adjuvant chemotherapy regimens in NCCN guideline.^13^ In order to improve the survival of patients with early NSCLC after a definitive surgery for resected lung tumors, we initiated this study to investigate whether patients with early‐stage and resected NSCLC without mediastinal lymph node involvement could benefit from adjuvant oral UFT or intravenous chemotherapy.

## 2 PATIENTS AND METHODS

### 2.1 Study design and population

The dataset we used is a population‐based registry system, Taiwan Cancer Registry (TCR), which includes over 90% of all cancer cases in Taiwan.^24^, ^25^, ^26^ We retrieved patients with early‐stage NSCLC identified by pathological confirmation, including those in stages IB and IIA (without any lymph node involvement) from the TCR during January 2011 and December 2017. The pathological TNM staging data of recruited cohort was adopted from the TCR dataset, and the 7th edition lung cancer staging system was used in this study.^27^ During 2010 and 2017, cancer stage (including lung cancer) at diagnosis in TCR followed the AJCC staging 7th edition per the requirement and regulations by TCR (https://twcr.tw/).

Patients were retrieved if they received systemic chemotherapy or oral UFT within 180 days after surgery. Patients who had chemotherapy prior to surgery, did not receive any systemic treatment after surgery, or had incomplete treatment information were excluded. In Taiwan, oral UFT for 2 years has also been reimbursed by the Taiwan National Health Insurance as adjuvant chemotherapy for at least T2 NSCLC, tumor size equal or greater than 3 cm, after complete tumor resection. Therefore, we focused that the comparison of adjuvant therapy effectiveness in early NSCLC patients receiving intravenous chemotherapy or oral UFT as treatment.

The enrolled population was identified, and their basic profile, underlying diseases, and use of chemotherapy or oral UFT were determined from the National Health Insurance Research Database (NHIRD) in Taiwan.^25^, ^26^, ^28^, ^29^ Meanwhile, the information of mortality was obtained from the Department of Statistics, Taiwan. Then, we investigated and analyzed the data and information of our longitudinal cohort by using the linkage between the two databases, TCR and NHIRD, till December 31, 2019.

### 2.2 Data collection and definition

The Eastern Cooperative Oncology Group (ECOG) scoring system was applied for evaluating patients' performance status.^30^ Additionally, we used the Charlson comorbidity index (CCI) from the NHIRD to assess our patients' comorbid conditions,^31^ but all malignancy‐associated scores should be not included (viewed as cancer‐free CCI) as previously presented.^24^, ^26^ In Taiwan's healthcare system, hospitals are classified into three levels based on their ability to provide care: medical centers, regional hospitals, and district hospitals. The ICD codes we used for identifying patients with adenocarcinoma or squamous cell carcinoma in the TCR and NHIRD were listed in Table S1. The adjuvant chemotherapy regimens per the regulations by Taiwan NHI included etoposide, gemcitabine, paclitaxel, docetaxel, and vinorelbine, combining with platinum agent or not, but pemetrexed was not included as adjuvant chemotherapy for early‐stage NSCLC patients after surgery in Taiwan (https://www.nhi.gov.tw/).

### 2.3 Statistical analysis

In order to describe the enrolled patients' characteristics, we used proportions or means for the distribution of the study population. The chi‐square test was used for assessing the association between categorical variables, and one‐way ANOVA or the Student's *t*‐test was used to analyze continuous variables. The cohort start date was recorded as the date of adjuvant therapy initiation. OS, the primary outcome, was defined as the duration from diagnosis to death for any cause. If the enrolled patients were alive at the time of the study's end (December 31, 2019), they were censored. The secondary outcome was relapse‐free survival (RFS), which was defined as the period from the cohort start date to the time of tumor recurrence (defined by either tumor recurrence from TCR, switching to systemic chemotherapy in patients with oral UFT, or initiation of 2nd line chemotherapy in patients receiving chemotherapy as adjuvant therapy) or death.

The propensity score (PS) estimated the probability of oral UFT or intravenous chemotherapy use by using a logistic regression analysis, and the potential confounders included age, gender, performance status, body mass index (BMI), tumor cell type, cancer stages, operation type, smoking and drinking habits, CCI, epidermal growth factor receptor (*EGFR*) mutation status, factors of patients at risk for relapse (high tumor grade, large tumor size, operation type, incomplete microscopic tumor margin resection, VPI, lymph node involvement, or mediastinal LAP sampling), and hospital levels. The ratio of matched cohorts of oral UFT and chemotherapy was set at 1:1, and the variables were balanced between the matched groups. If variables between matched cohorts remained unbalanced, all variable were further adjusted in the final model. The caliper distance and standardized mean differences before and after PS matching analysis, for each variable, have been provided in Table S2. After matching, we further performed the subgroup analyses among the different groups, including age, BMI, ECOG, gender, smoking and drinking habit, stages (IB and IIA), *EGFR* mutation status, tumor size, and tumor with VPI. The SAS software (version 9.4; SAS Institute Inc.) was used to perform all analyses. A statistical result was viewed as significant at a *p* value less than 0.05 on a two‐sided test.

## 3 RESULTS

### 3.1 Patient characteristics

**FIGURE 1:**
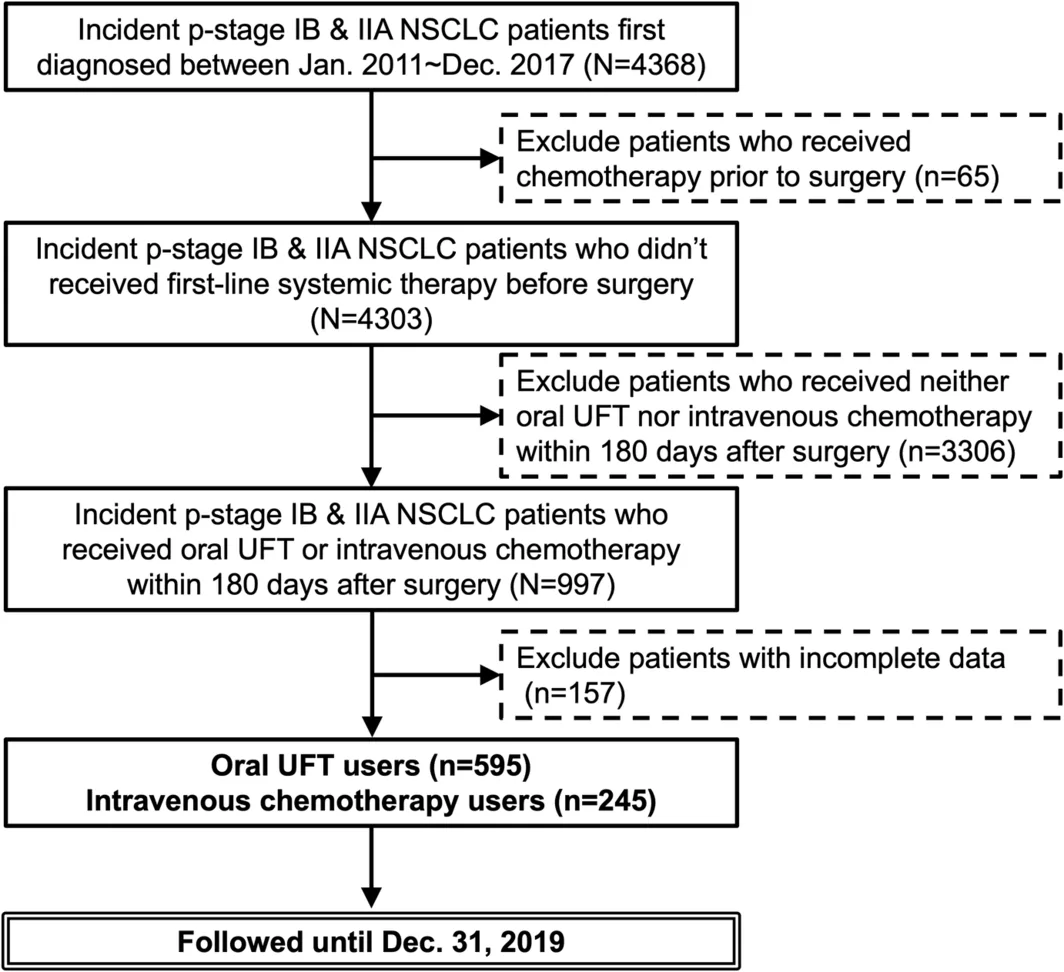
Flow chart of patient recruitment. NSCLC, non‐small cell lung cancer; UFT, uracil‐tegafur.

Between January 2011 and December 2017, we retrieved 4368 NSCLC patients with pathologic stage IB and IIA (AJCC 7th edition) and without N1 lymph node involvement. Among them, a total of 840 patients with early‐stage NSCLC receiving adjuvant therapy after definitive surgery (including 595 oral UFT and 245 intravenous chemotherapy) were finally enrolled (Figure 1). The clinical characteristics of the enrolled patients were summarized in Table 1. The median age of all patients was 65 years, and the majority was female (*n* = 444, 52.9%). Most of our enrolled patients had a relatively good performance status (ECOG ≦1: *n* = 818, 97.4%) and had never smoked (*n* = 545, 64.9%) and were nondrinkers (*n* = 643, 76.6%). Meanwhile, 735 (87.5%) patients were adenocarcinoma, and 737 (87.7%) patients had stage IB disease (Table 1).

**TABLE 1:** Characteristics of early‐stage NSCLC patients receiving surgery.

	Unmatched			*p* ^a^	Matched			*p* ^a^
	All (*N* = 840)	Oral UFT (*N* = 595)	Intravenous CT (*N* = 245)		All (*N* = 352)	Oral UFT (*N* = 176)	Intravenous CT (*N* = 176)	
Age	64.4 ± 0.8	64.9 ± 9.8	63.1 ± 9.8	0.0131	63.3 ± 9.9	63.3 ± 10.2	63.5 ± 9.6	0.8973
<45 year	22 (2.6)	12 (2.0)	115 (46.9)	0.0980	178 (50.6)	93 (52.8)	85 (48.3)	0.6251
45–65 year	388 (46.2)	268 (45)	120 (49.0)		161 (45.7)	76 (43.2)	85 (48.3)	
>65 year	430 (51.2)	315 (52.9)	10 (4.08)		13 (3.69)	7 (3.98)	6 (3.41)	
Male	396 (47.1)	260 (43.7)	136 (55.5)	0.0018	168 (47.7)	84 (47.7)	84 (47.7)	>0.9999
BMI	24.2 ± 3.9	24.2 ± 4.0	24.1 ± 3.7	0.6359	23.9 ± 3.8	23.8 ± 3.7	24.0 ± 3.8	0.5690
<18	32 (3.81)	25 (4.2)	7 (2.9)	0.2888	13 (3.7)	6 (3.4)	7 (4.0)	0.8049
18–24	393 (46.8)	269 (45.2)	124 (50.6)		182 (51.7)	94 (53.4)	88 (50)	
>24	415 (49.4)	301 (50.6)	114 (46.5)		157 (44.6)	76 (43.2)	81 (46.0)	
ECOG ≦1	818 (97.4)	582 (97.8)	236 (96.3)	0.2373	342 (97.2)	172 (97.7)	170 (96.6)	0.7503
Never smoker	545 (64.9)	414 (69.6)	131 (53.5)	<0.0001	214 (60.8)	108 (61.2)	106 (60.2)	0.8772
Nondrinker	643 (76.6)	464 (78.0)	179 (73.1)	0.1287	265 (75.3)	130 (73.9)	135 (76.7)	0.6213
CCI score	0.70 ± 1.44	0.71 ± 1.49	0.68 ± 1.32	0.7909	0.69 ± 1.46	0.69 ± 1.51	0.69 ± 1.40	>0.9999
Cell type								
Adenocarcinoma	735 (87.5)	559 (93.9)	176 (71.8)	<0.0001	296 (84.1)	149 (84.7)	147 (83.5)	0.8843
Squamous cell carcinoma	105 (12.5)	36 (6.05)	69 (28.2)		56 (16.0)	27 (15.3)	29 (16.5)	
Staging								
IB	737 (87.7)	573 (96.3)	164 (66.9)	<0.0001	306 (86.9)	154 (87.5)	152 (86.4)	0.7518
IIA	103 (12.3)	22 (3.7)	81 (33.1)		46 (13.1)	22 (12.5)	24 (13.6)	
*EGFR* status								
Mutant	221 (26.3)	172 (28.9)	49 (20.0)	0.0179	85 (24.2)	40 (22.7)	45 (25.6)	0.3937
Wild type	122 (14.5)	79 (13.3)	43 (17.6)		55 (15.6)	24 (13.6)	31 (17.6)	
Unknown	497 (59.2)	344 (57.8)	153 (62.4)		212 (60.2)	112 (63.6)	100 (56.8)	
Hospital level								
Medical center	631 (75.1)	484 (81.3)	147(60.0)	<0.0001	216 (61.4)	106 (60.2)	110 (62.5)	0.7427
Risk factors for recurrence								
1. Tumor size								
≦4 cm	549 (65.4)	432 (72.6)	117 (47.8)	<0.0001	223 (63.4)	114 (64.8)	109 (61.9)	0.5802
>4 cm	291 (34.6)	163 (27.4)	128 (52.2)		129 (36.7)	62 (35.2)	67 (38.1)	
2. Tumor grade								
Grade 1 and 2	595 (70.8)	429 (73.8)	156 (63.6)	0.0044	238 (67.6)	117 (66.5)	121 (68.8)	0.6487
Grade 3 and 4	245 (29.2)	146 (26.2)	89 (36.3)		114 (32.4)	59 (33.5)	55 (31.3)	
3. Operation method								
Lobectomy	749 (89.2)	539 (90.6)	210 (85.7)	0.0759	300 (85.2)	155 (88.1)	145 (82.4)	0.2717
Wedge resection	71 (8.5)	42 (7.06)	29 (11.8)		44 (12.5)	17 (9.66)	27 (15.4)	
Others	20 (2.38)	14 (2.35)	6 (2.45)		8 (2.27)	4 (2.27)	4 (2.27)	
4. With visceral pleural invasion	491 (58.3)	333 (56.0)	158 (64.7)	0.0023	228 (64.8)	113 (64.2)	115 (65.3)	0.8234
Patients with any above risk factors	696 (82.9)	471 (79.2)	225 (91.8)	<0.0002	312 (88.6)	156 (88.6)	156 (88.6)	>0.9999

*Note*: Data are mean ± standard deviation, or number (%).

Abbreviations: BMI, body mass index; CCI, Charlson comorbidity index; CT, chemotherapy; ECOG, Eastern Cooperative Oncology Group; EGFR, epidermal growth factor receptor; SD, standard deviation; UFT, uracil‐tegafur.

^a^ Comparing between two groups (UFT and chemotherapy).

Considering the comparison between oral UFT and chemotherapy users' characteristics, oral UFT users were significantly older (64.9 ± 9.8 vs. 63.1 ± 9.8 years, *p* = 0.013) and more likely to be female patients (444, 52.9%, *p* = 0.018) than patients receiving intravenous chemotherapy. Adjuvant intravenous chemotherapy agents included 159 patients (64.9%) using vinorelbine, 57 (23.3%) gemcitabine, 16 (6.5%) docetaxel, 8 (3.3%) etoposide, and 5 (2.0%) paclitaxel, and there were 170 of 245 patients (69.4%) receiving platinum‐based chemotherapy as treatment regimen (Figure S1). Furthermore, the median treatment length in UFT, platinum doublet chemotherapy and non platinum‐based chemotherapy was 19.8, 2.6 and 3.3 months respectively (Table 2). Meanwhile, UFT users frequently were never smokers, adenocarcinoma, pathologic stage IB disease, mutant *EGFR*, and at medical center for treatment (Table 1). For all of the enrolled participants after curative surgery, 98.9% of patients had complete microscopic tumor resection (R0) and 99.3% of patients received mediastinal lymph node sampling. Patients receiving intravenous chemotherapy as adjuvant therapy significantly meet the identified risk factors for tumor recurrence, including large tumor size (>4 cm), high tumor grade (grade 3 or 4), and VPI. However, the operation types (lobectomy or wedge resection) are not different between oral UFT or chemotherapy users (Table 1).

**TABLE 2:** The treatment length of adjuvant therapy.

	Unmatched			Matched		
	Oral UFT (*N* = 595)	Platinum doublet chemotherapy (*N* = 170)	Non platinum‐based chemotherapy (*N* = 75)	Oral UFT (*N* = 176)	Platinum doublet chemotherapy (*N* = 118)	Non platinum‐based chemotherapy (*N* = 58)
Treatment length, months	15.6 ± 10.1 (19.8)	2.9 ± 2.4 (2.6)	3.3 ± 2.7 (3.4)	14.5 ± 10.1 (15.9)	2.9 ± 2.0 (2.4)	3.2 ± 2.7 (3.2)

*Note*: Data are mean ± standard deviation (median).

Abbreviation: UFT, uracil‐tegafur.

### 3.2 OS and RFS of early NSCLC patients receiving oral UFT or chemotherapy as adjuvant therapy in the unmatched cohort

**FIGURE 2:**
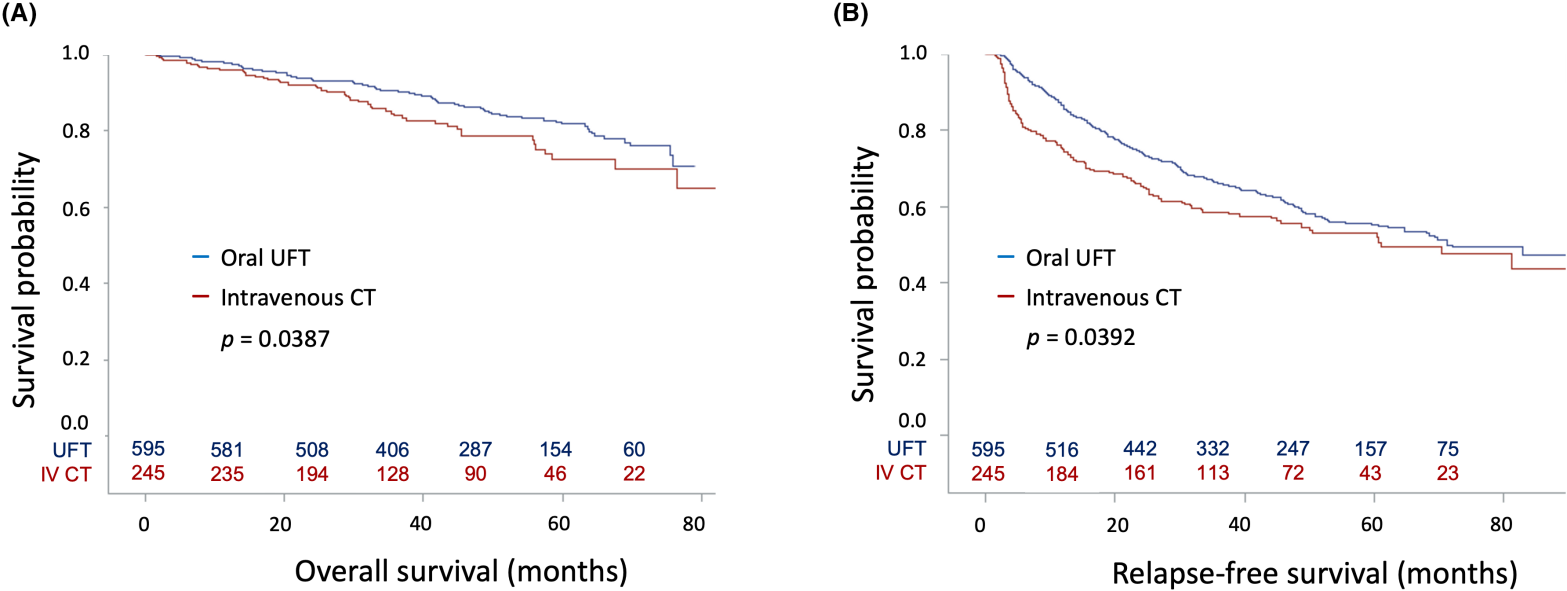
Kaplan–Meier curves for overall survival (OS) and relapse‐free survival (RFS) according to patients using oral uracil‐tegafur (UFT) and intravenous chemotherapy (CT) in overall cohort. (A) Kaplan–Meier curves for OS between oral UFT and intravenous CT users; (B) Kaplan–Meier curves for RFS between UFT and intravenous CT users.

The Kaplan–Meier curve of OS in patients receiving oral UFT or intravenous chemotherapy as adjuvant therapy is illustrated in Figure 2A. Although the mortality events among all patients were less than 50%, patients using oral UFT had significantly longer OS than those with intravenous chemotherapy (HR: 0.69, 95% CI: 0.49–0.98, *p* = 0.0387, Figure 2A). The RFS (mean ± SD, months) (41.2 ± 23.0 vs. 36.0 ± 25.4) of the oral UFT groups was also longer than intravenous chemotherapy group (HR: 0.79, 95% CI: 0.61–0.97, *p* = 0.0392) (Figure 2B).

### 3.3 Comparing the OS and RFS of matched oral UFT and chemotherapy users

**FIGURE 3:**
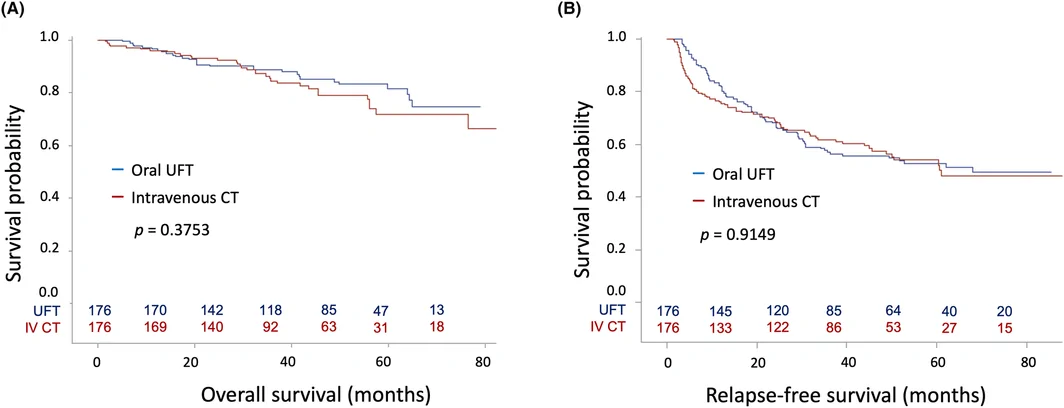
Kaplan–Meier curves for overall survival (OS) and relapse‐free survival (RFS) according to patients using oral uracil‐tegafur (UFT) and intravenous chemotherapy (CT) in matched cohorts. (A) Kaplan–Meier curves for OS between matched oral UFT and intravenous CT users; (B) Kaplan–Meier curves for RFS between matched oral UFT and intravenous CT users.

In the PS matched cohort, a matched cohort of 176 oral UFT users and 176 chemotherapy users was assembled and created by balancing the clinical variables as much as possible. In the Cox regression analysis, long‐term OS was not different between oral UFT and chemotherapy group (oral UFT vs. intravenous chemotherapy, HR: 0.80, 95% CI: 0.48–1.32, *p* = 0.3753, Figure 3A). Also, RFS (mean ± SD, months) (35.1 ± 23.9 vs. 36.0 ± 25.2) was not different between patients with oral UFT and intravenous chemotherapy group (oral UFT vs. intravenous chemotherapy, HR: 0.98, 95% CI: 0.72–1.34, *p* = 0.9149) (Figure 3B).

### 3.4 Matched subgroups analysis of oral UFT versus conventional chemotherapy

**FIGURE 4:**
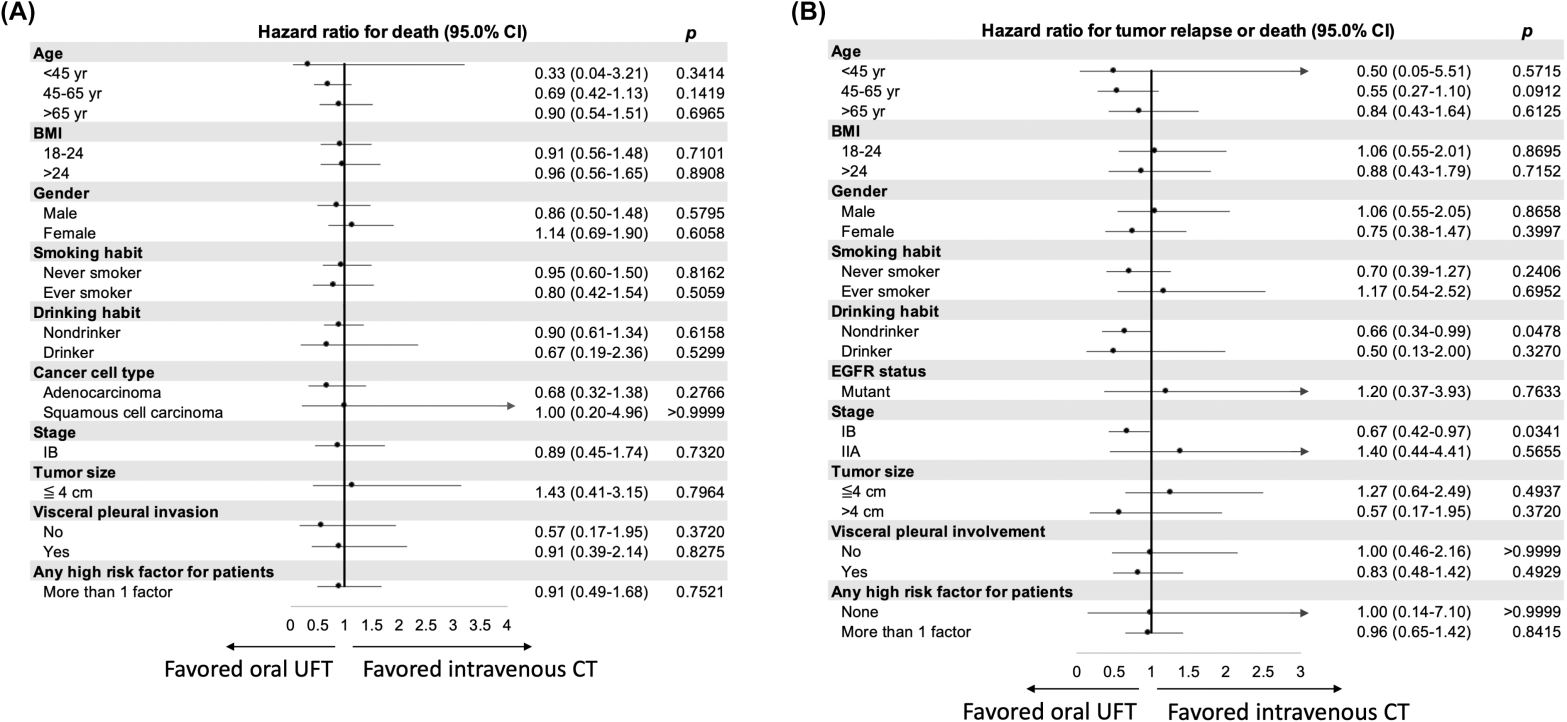
Forest plot for the matched subgroup analysis on (A) overall survival (OS) and (B) relapse‐free survival (RFS). Subgroup analysis of age less than 45 years old, body mass index (BMI) less than 18, EGFR status, stage IIA as well as tumor size more than 4 cm in OS and age less than 45 years old, BMI less than 18 as well as histologic type in RFS was not performed because iteration limit reached without convergence owing to limited data sample. CT, chemotherapy; EGFR, epidermal growth factor receptor; UFT, uracil‐tegafur.

Forest plots were used for comparing OS and RFS between PS matched subgroup patients receiving oral UFT and intravenous chemotherapy as adjuvant therapy, as illustrated in Figure 4A,B, respectively. Early‐stage NSCLC patients with oral UFT and intravenous chemotherapy as adjuvant therapy could provide similar OS benefits among all subgroups (Figure 4A). It is worthy to note that early‐stage NSCLC patients with oral UFT use reached a significant RFS benefit among the subgroups of non‐drinkers (oral UFT vs. intravenous chemotherapy, HR: 0.66, 95% CI: 0.34–0.99, *p* = 0.0478) and patients with stage IB disease (oral UFT vs. intravenous chemotherapy, HR: 0.67, 95% CI: 0.42–0.97, *p* = 0.0341) (Figure 4B).

## 4 DISCUSSION

Our study found that oral UFT as adjuvant therapy was not different from intravenous chemotherapy in terms of OS and RFS in early‐stage NSCLC patients without mediastinal lymph node involvement (stage IB and IIA) after complete tumor resection. Among the elderly NSCLC population with stage IB and IIA disease receiving oral UFT as adjuvant therapy, oral UFT was not associated with a significant OS or RFS benefit over IV chemotherapy. From the subgroup analyses of our study, there was no significant RFS and OS difference in age, sex, BMI, and smoking habit, between postoperative oral UFT and intravenous chemotherapy users. Remarkably, patients with stage IB or non‐drinking NSCLC receiving oral UFT as adjuvant therapy were associated with longer RFS than intravenous chemotherapy group.

Two earlier prospective trials reported a survival benefit of adjuvant oral UFT treatment in patients with stage IB adenocarcinoma and stage I to III NSCLC.^6^, ^19^ A recent retrospective real‐world study in Japan demonstrated that the use of oral UFT after complete resection of stage I NSCLC could improve OS and RFS, especially in patients aged 45 to 75 years, and the results were consistent with previous clinical trials.^32^ However, the evidence of comparing adjuvant oral UFT with adjuvant intravenous chemotherapy for stage I to IIIA NSCLC remained limited, and the number of cases in these trials was small.^6^, ^19^ Additionally, a previous clinical trial using paclitaxel plus carboplatin as an experimental regimen did not show an advantage as adjuvant therapy in patients with stage IB disease.^9^ Recently, another study also used the same regimen of paclitaxel plus carboplatin compared with UFT for patients with stage IB to IIIA NSCLC who underwent complete surgical resection, but this regimen was not better than UFT in terms of survival.^33^ Our study results were consistent with previous clinical trials, and we observed a trend toward a potential benefit in RFS for patients with stage IB NSCLC who received oral UFT as adjuvant therapy. Additionally, our study presents several key differences compared to Toyooka et al.'s clinical trial. We utilized a population‐based approach using data from the TCR, providing a broader representation of stage IB‐IIA NSCLC patients in real‐world clinical practice, and our study contributed to the literature by examining the effectiveness of different adjuvant chemotherapy options, accommodating patients with weaker or poorer performance often excluded from clinical trials. Although there was 1 cm change in T2a (>3 cm–5 cm) from 7th edition lung cancer staging system to 8th edition, stage IB lung cancer disease in AJCC 7th edition is broadened to stage IB (T2a, >3–4 cm) & IIA (T2b, >4–5 cm) disease in AJCC 8th edition.^7^, ^27^ Meanwhile, our study still indicated that UFT showed favorable trend in RFS for patients with tumor less than 4 cm by our matched subgroup analysis. Specifically, UFT as adjuvant therapy could be a comparable option for patients with early‐stage and resected NSCLC, in line with conventional intravenous chemotherapy, even when considering the AJCC 8th edition lung cancer staging system.

The adverse effects of long‐term and even high‐dose use of UFT are mild, and most common adverse effects were gastrointestinal symptoms, such as nausea, vomiting, and diarrhea.^19^, ^34^ In our study, UFT users in the unmatched and matched cohort had median treatment duration 19.8 and 15.9 months, respectively (Table 2). Meanwhile, patients receiving platinum doublet chemotherapy in either unmatched or matched cohort had shorter treatment duration than non platinum‐based chemotherapy, and the intolerance of platinum chemotherapy related toxicity to patients may cause the difference in treatment course. However, UFT related liver damage, including elevated bilirubin and liver enzymes, is another frequent and important toxic reaction.^19^, ^34^ Our study found that oral UFT use among nondrinkers have longer RFS than those receiving intravenous chemotherapy via matched subgroup analysis. In vitro study, cytochrome P450 CYP2A6 is the key enzyme in the metabolism of tegafur to 5‐FU,^35^ and alcohol consumption could induce the expression of CYP2A6 of innate immune cells causing oxidative stress, cytotoxic effect, and decreased drug efficacy.^36^ Therefore, the effects and side effects of UFT use as adjuvant therapy for resected NSCLC in drinkers should be carefully monitored.

According to the NCCN guideline for NSCLC management,^13^ there are some identified high risk factors for tumor recurrence, including poorly differentiated tumors, vascular invasion, large tumor size (>4 cm), undergoing wedge resection, VPI and unknown lymph node status. While lobectomy is considered the standard surgical treatment for early‐stage lung cancer in Taiwan (with a percentage of 89.2% in our study), sublobar resection, including segmentectomy or wedge resection, is being increasingly recognized as a viable alternative in certain cases.^37^, ^38^ This approach may be considered for patients with smaller tumors located in peripheral areas of the lung, as well as those who have poor pulmonary function, other major comorbidities, or advanced age, which may contraindicate lobectomy. Therefore, this real‐world study directly compared the effectiveness of adjuvant oral UFT and intravenous chemotherapy for patients with early‐stage resected NSCLC, and all risk factors were carefully included as well as adjusted in this study.

Meanwhile, around 20%–30% of our enrolled patients were found to have *EGFR* mutations in the cancer registry, but no significant difference was found in the RFS of patients receiving adjuvant UFT or chemotherapy. The use of EGFR TKIs as adjuvant therapy in early‐stage NSCLC patients with *EGFR* mutations is not recommended by the NCCN guideline,^13^ but several studies recently reported that EGFR TKIs could prolong RFS in stage II and stage IIIA patients compared to those receiving conventional intravenous chemotherapy^39^, ^40^, ^41^ However, the benefit of OS for patients with *EGFR* mutant NSCLC who receive TKI as adjuvant therapy after complete surgical resection is still being studied. Additionally, one recent phase 3 trial demonstrated that adjuvant atezolizumab, an immune checkpoint inhibitor, following the platinum‐based chemotherapy could provide disease‐free survival benefit among stage II–IIIA NSCLC patients and specific subgroup of tumors expressing PD‐L1 ≥1%,^42^ and further immunotherapy, including anti‐PD‐1 and anti‐PD‐L1 agents, is being investigated as adjuvant therapy for patients with resected stage IB to IIIA NSCLC to evaluate the clinical benefit in RFS.^43^

Some limitations of our study should be mentioned. First, this is an observational real‐world study, and confounding by indication is still a concern in our study while chemotherapy may still be preferred among patients with a higher risk for recurrence. While we have tried to control for potential confounders by PS matching, the possibility of unmeasured confounders may exist. We, therefore, took a more conservative viewpoint toward data interpretation in this article. Meanwhile, the results of subgroup analyses should be interpreted with caution due to the absence of adjustment for multiplicity in this study. Second, treatment‐related toxicity profiles were hard to ascertain in this dataset. In treating patients, oral UFT was known to be less toxic than newer generation chemotherapy. Despite our efforts to adjust for potential confounding factors and utilize PS matching to minimize differences between the two treatment groups, it is important to acknowledge the presence of residual confounding factors. Our study was, however, unable to evaluate oral UFT and intravenous chemotherapy related side effects and elucidate whether these side‐effects would alter the severe infection rate, quality of life, and treatment course completion rate to further influence the clinical outcomes. Furthermore, we did not include a control group, among whom did not receive adjuvant therapy in this study. The reasons to decline adjuvant therapy can be diverse and hard to be identified in the claims database. We, therefore, consider that including such a control group would lead to the introduction of significant bias that could not be adequately adjusted.

In conclusion, despite the fact that adjuvant oral UFT is not currently recommended as a treatment option for early‐stage NSCLC management in the NCCN guideline, our study suggests that patients using oral UFT as adjuvant intravenous chemotherapy may achieve similar RFS and OS. Furthermore, oral UFT administration was associated with a longer RFS than intravenous chemotherapy in patients with stage IB (AJCC 7th edition, equivalent to stage IB and IIA by AJCC 8th edition) disease who were nondrinkers.

## AUTHOR CONTRIBUTIONS

**Sheng‐Kai Liang:** Conceptualization (equal); data curation (equal); formal analysis (lead); funding acquisition (equal); investigation (equal); writing – original draft (lead). **Chang‐Wei Wu:** Data curation (equal); resources (equal). **Ching‐I Chang:** Data curation (equal); resources (equal). **Li‐Ta Keng:** Data curation (equal); funding acquisition (equal); investigation (equal). **Meng‐Rui Lee:** Conceptualization (equal); funding acquisition (lead); investigation (equal); supervision (lead); writing – original draft (equal); writing – review and editing (equal). **Jann‐Yuan Wang:** Investigation (equal); validation (equal); writing – review and editing (equal). **Jen‐Chung Ko:** Writing – review and editing (equal). **Wei‐Yu Liao:** Validation (equal); writing – review and editing (equal). **Kuan‐Yu Chen:** Validation (equal); writing – review and editing (equal). **Chao‐Chi Ho:** Validation (equal); writing – review and editing (equal). **Jin‐Yuan Shih:** Supervision (equal); validation (equal); writing – review and editing (equal). **Chong‐Jen Yu:** Supervision (equal); writing – review and editing (equal).

## FUNDING INFORMATION

This work was founded by a grant from the National Taiwan University Hospital Hsinchu Branch (grant no. 110‐HCH‐017).

## CONFLICT OF INTEREST STATEMENT

S.K.L. has received speaking honorarium from Boehringer Ingelheim, AstraZeneca, Pfizer, and Merck Sharp & Dohme. M.R.L. and J.Y.W. have received speaking honorarium from Pfizer, Roche, and Daiichi Sankyo. K.Y.C. has received honoraria for speech from Pfizer, Chugai Pharmaceutical, Novartis, Eli Lilly, AstraZeneca, Roche, Merck Sharp & Dohme, Boehringer Ingelheim, and Bristol‐Myers Squibb, as well as travel/accommodation/meeting expenses from Chugai Pharmaceutical, Merck Sharp & Dohme, and Boehringer Ingelheim. C.C.H. has received honorarium for speech or participated in compensated advisory board: Boehringer Ingelheim, Genentech, Chugai, Eli Lilly, Amgen, Roche, Novartis, BMS, MSD, Pfizer, and Ono pharmaceutical. J.Y.S. has served as an advisory board member from Roche, Boehringer Ingelheim, Amgen, AstraZeneca, Eli Lilly, Merck Sharp & Dohme, Chugai Pharma, Pfizer, Takeda, CStone Pharmaceuticals, Novartis, Ono Pharmaceutical, Janssen, and Bristol‐Myers Squibb; received speaking honoraria from Genconn Biotech, AstraZeneca, ACTgenomics, Amgen, Roche, Eli Lilly, Pfizer, Novartis, Bayer, Boehringer Ingelheim, Merck Sharp & Dohme, Chugai Pharma, CStone Pharmaceuticals, Janssen, Takeda, TTY Biopharm, MundiPharma, Ono Pharmaceutical, Orient EuroPharma, and Bristol‐Myers Squibb; received support for attending meetings from Roche, Boehringer Ingelheim, AstraZeneca, and Chugai Pharma; as well as grant from Roche. C.W.W., C.I C., L.T.K., J.C.K., W.Y.L., and C.J.Y. have no relevant conflicts of interest. to declare.

## ETHICS STATEMENT

This study was approved by the Institutional Review Board (IRB) committee of the National Taiwan University Hospital Hsinchu Branch (NTUH‐HC REC: 109‐070‐E). All methods we used were carried out following the principles of the Declaration of Helsinki.

## PATIENT CONSENT STATEMENT

The requirement for informed consent from all research participants was waived by the Institutional Review Board committee because all the data and information we utilized were de‐identified.

## Supporting information


Figure S1.

Table S1.

## Data Availability

This article and its Supporting Information contain all of the data presented in this study.
