# Supplementary figures and images for: Oral uracil–tegafur compared with intravenous chemotherapy as adjuvant therapy for resected early‐stage non‐small cell lung cancer patients
Source: Cancer Med. 2023 Aug 9;12(17):17993–8004. doi: 10.1002/cam4.6440 (PMC10523960; doi:10.1002/cam4.6440)

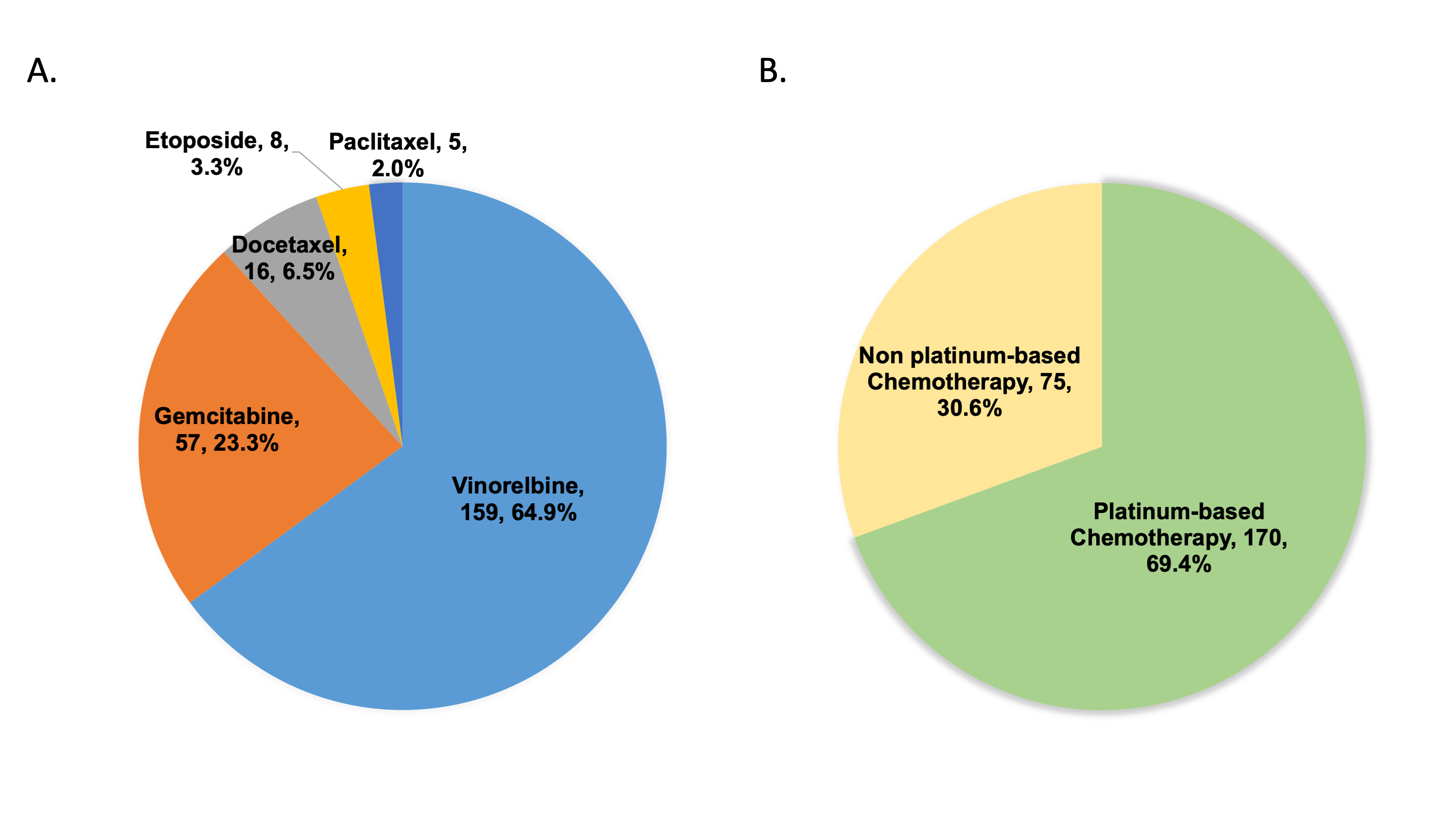

Supplement: Supplementary file 1 — Figure S1. [file CAM4-12-17993-s002.tiff]
