# Supplementary material for: Oral uracil–tegafur compared with intravenous chemotherapy as adjuvant therapy for resected early‐stage non‐small cell lung cancer patients
Source: Cancer Med. 2023 Aug 9;12(17):17993–8004. doi: 10.1002/cam4.6440 (PMC10523960; doi:10.1002/cam4.6440)
Supplement: Supplementary file 2 — Table S1. [file CAM4-12-17993-s001.docx]

**Supplementary Table S1. Defining codes in this study for Taiwan Cancer Registry and National Health Insurance database**

| **Cell types** | **International Classification of Diseases for Oncology, 3rd edition (ICD-O-3) code for lung site (C340-C343, C348-C349)** |
| --- | --- |
| Lung adenocarcinoma | 8140, 8141, 8143, 8147, 8200, 8201, 8250~8255, 8260, 8310, 8320, 8323, 8480, 8481, 8490 |
| Squamous cell carcinoma | 8052, 8070, 8072, 8073, 8074, 8083, 8084 |

**Supplementary Table S2. Caliper distance and standardized mean differences for each variable before and after propensity score matching**

|  |  | Pre matching | |  | Post matching | |
| --- | --- | --- | --- | --- | --- | --- |
|  |  | Caliper distance | standard mean difference |  | Caliper distance | standard mean difference |
| **Age** |  | 5.366 | -0.188 |  | 3.070 | 0.014 |
| **Male** |  | 0.273 | 0.496 |  | 0.155 | 0.000 |
| **BMI** |  | 2.117 | -0.035 |  | 1.174 | 0.021 |
| **ECOG≦1** |  | 0.016 | -0.017 |  | 0.009 | 0.000 |
| **Never smoker** |  | 0.264 | -0.340 |  | 0.152 | 0.023 |
| **Nondrinker** |  | 0.571 | -0.124 |  | 0.351 | -0.111 |
| **CCI score** |  | 0.774 | -0.017 |  | 0.452 | 0.000 |
| **Cell type** |  | 0.198 | 0.668 |  | 0.114 | 0.031 |
| **Staging** |  | 0.197 | -0.829 |  | 0.105 | 0.034 |
| ***EGFR* status** |  | 0.235 | 0.212 |  | 0.133 | 0.047 |
| **Hospital level** |  | 0.244 | -0.476 |  | 0.151 | -0.047 |
| **Risk factors for recurrence** |  |  |  |  |  |  |
| 1.Tumor size |  | 0.261 | -0.532 |  | 0.150 | 0.059 |
| 2.Tumor grade |  | 0.254 | -0.239 |  | 0.145 | -0.048 |
| 3.Operation method |  | 0.227 | 0.123 |  | 0.134 | 0.131 |
| 4.With visceral pleural invasion |  | 0.060 | 0.187 |  | 0.033 | 0.000 |
| **Patients with any above risk factors** |  | 0.439 | 0.570 |  | 0.241 | 0.037 |

Abbreviation: BMI, body mass index; CCI, Charlson comorbidity index; ECOG, Eastern Cooperative Oncology Group; EGFR, epidermal growth factor receptor.
